# Standing tall or falling short: A narrative review of height dissatisfaction and psychological outcomes

**DOI:** 10.1371/journal.pmen.0000497

**Published:** 2025-11-18

**Authors:** Nathaniel P. Schmidt, Daniel Talbot

**Affiliations:** School of Behavioural and Health Sciences, Australian Catholic University, Sydney, Australia; PLOS: Public Library of Science, UNITED KINGDOM OF GREAT BRITAIN AND NORTHERN IRELAND

## Abstract

Height is a highly visible and socially significant physical characteristic that influences numerous psychosocial outcomes, yet height dissatisfaction - negative evaluation of one’s own height - remains an underexplored dimension of body image research. Our narrative review synthesizes the current scope of literature on height and height dissatisfaction, highlighting their associations with psychological correlates such as self-esteem, body image concerns, and mental health symptoms including anxiety and depression. Theoretical perspectives from evolutionary psychology and sociocultural frameworks, including the Tripartite Influence Model, were applied to elucidate potential origins and maintenance of height dissatisfaction. While empirical research remains limited, particularly, regarding clinical populations and female height dissatisfaction, emerging findings underscore the complex interplay between biological, psychological, and social factors of height. Recommendations for future research emphasize the need for more comprehensive and validated measures of height dissatisfaction that capture both desires to be taller or shorter, as well as related cognitive, affective, and behavioral components. Our review aims to advance understanding of height dissatisfaction as a unique aspect of body image, with important implications for clinical practice and psychosocial interventions.

## Introduction

Height is a fundamental and socially salient physical attribute that influences body image, self-esteem, and a range of psychological and social outcomes. Unlike other aspects of body image, such as muscularity or body fat, height cannot be meaningfully altered without invasive or risky medical interventions [1]. For men, taller stature is associated with perceptions of greater masculinity and dominance [2], as well as traits like strength and power [3]. Height also plays a critical role in social and romantic contexts, contributing to perceived attractiveness and suitability as a partner [4,5]. For example, Salska, Frederick [3] found that only 23% of heterosexual men and 4% of heterosexual women would accept a relationship in which the woman was taller, highlighting the persistent gendered norms surrounding height. For women, cultural stereotypes often associate femininity with smaller stature and finer bone structure [6]. However, research also shows that women may express a desire to be taller [7], suggesting that height dissatisfaction is not exclusively a male concern.

Height dissatisfaction refers to the negative evaluation of one’s own height, whether feeling too short or too tall [6]. This form of body dissatisfaction has been linked to a range of negative psychological outcomes, including reduced wellbeing [8], lower quality of life [7], and psychopathology such as disordered eating [9]. Despite these associations, height and height dissatisfaction remain significantly under-researched in clinical psychology.

Given the cultural importance placed on height for both men and women - and the potential consequences for psychological wellbeing - our review synthesizes current research on height, height dissatisfaction, and associated psychological and social outcomes. First, the review explores the relationship between height, height dissatisfaction, wellbeing, and social functioning. Next, it considers theoretical frameworks that explain height-related concerns. Finally, it discusses clinical implications and offers recommendations for future research and practice.

## Conceptualizing height and height dissatisfaction

Human height is an intricate trait, influenced by a number of genetic and environmental factors. Research has indicated that around 80% of variation in height is potentially attributable to genes [10,11], making the heritability of height rather high compared to other intricate traits [12] such as weight [13]. Additionally, differences in growth curves have been observed across ethnic groups, for example, African-origin populations in the USA and Europe have a higher mean body height when compared to their white counterparts [14]. While this suggests that height is substantially influenced by genes, there has also been a notable increase in mean height at a population level across the last 150 years [15]. In Western countries, this increase in mean height likely reflects the influence of environmental factors such as increases in health care quality, education, nutrition, and social equality [16].

Height dissatisfaction is a psychological aspect of height. It pertains to negative perception (e.g., I am too short) and affective experiences (e.g., low mood due to short stature) relating to one’s own height, as well as related behaviors (e.g., seeking ways to grow taller) [17]. Typically, height dissatisfaction reflects a discrepancy between a person’s actual height and their perceived height ideal. This notion is further reflected by research that height and height dissatisfaction are negatively correlated [18], meaning that generally, shorter people are more dissatisfied with their height.

## Height and men

Perceptions of what is an acceptable or attractive height are shaped by cultural and social norms. For men, masculine norms reflect a dominant set of traits that define what it means to be male within a particular cultural or societal context [19]. In Western cultures, the ideal or cultural norm is for men to be tall [20], and subsequently men are more concerned with their height and how it may be perceived. Social perceptions further amplify these concerns by associating taller men with traits such as dominance [2], assertiveness [21], and greater physical attractiveness compared to men of average or shorter height [22]. For instance, Ridgeway and Tylka [4] asked a sample of college men to describe their ideal male body, with height emerging as a critical component of this, further emphasizing this “tall, dark and handsome” notion amongst men. It seems that for men, height dissatisfaction is unidirectional; men who are dissatisfied with their height want to be taller.

## Height and women

For women, height has been less of a consideration in past research. One line of thinking proposes that female-related height concerns work in the opposite way to men; women do not want to be ‘too tall’ [6]. This perspective is rooted in societal ideals of femininity, which often emphasize delicacy, including a smaller frame and shorter stature [23]. It may also reflect a contrast to cultural associations of tallness with masculinity, where greater height is viewed as a symbol of male strength and dominance [2]. Again, this is reinforced by social norms in heterosexual romantic relationships consisting of a taller man and shorter woman [24]. At its extreme this preference for shorter female height has previously manifested in hormonal intervention [25]. In an older study, Barnard, Scialli [26] revealed that one-third of pediatric endocrinologists in the United States had offered growth suppression to taller stature girls as a treatment option in the five years preceding their study.

However, empirical evidence supporting the notion that women are dissatisfied with being too tall remains limited. In fact, existing studies suggest, similar to men, most women who report height dissatisfaction typically wish to be taller, rather than shorter. For instance, Jacobi and Cash [27] found women’s height ideals were significantly taller than their reported height. This was supported by Perkins, Hayes [7] who noted that 61% of their sample reported idealizing a height that differed from their own, and of this, 45% reported wanting to be taller. Additionally, Talbot and Mahlberg [6] found that 33% of women in their sample wanted to be taller, compared to just 16% who wanted to be shorter.

Theoretically, height dissatisfaction among women may be bidirectional: many women may desire to be taller, but not too tall. However, the existing empirical literature predominantly supports the former, with strong evidence indicating that women who are dissatisfied with their height generally wish to be taller.

## Measuring height dissatisfaction

Currently, height dissatisfaction is measured through two methods: (i) collecting respondents' actual height and ideal height (i.e., the height they wish they were), and calculating a discrepancy score between the two measures [6,18], and (ii) self-report scales. To the author’s knowledge there are only three published specific measures of height dissatisfaction: the Male Body Attitudes Scale (MBAS) [28], the Male Body Attitudes Scale-Revised (MBAS-R) [29] and Negative Physical Self Scale-Short (NPSS-S) [17].

The MBAS-height subscale consists of two items: “I am satisfied with my height” (reverse scored), and “I wish I were taller”, with higher scores indicating greater height dissatisfaction. However, the validity of this subscale has been questioned due to its brevity, as factors with fewer than three items are generally considered weak or unstable [30,31].

The MBAS-R-height subscale included an additional item “I feel ashamed of my height”, with higher scores once again indicating more prevalent levels of height dissatisfaction. It is important to note that the MBAS and revised version were designed for men, and validated using male samples, and as such their suitability for assessing height dissatisfaction amongst women is questionable. Additionally, the MBAS assumes height dissatisfaction only occurs in the direction of wanting to be taller, and does not accommodate taller individuals who are dissatisfied with their height and may wish to be shorter.

The NPSS-S shortness concern scale consists of 11 items relating to an individual’s perception of their height (“I think I am too short”) and their engagement in height related compensatory behaviors (“I keep trying ways to get taller”). Higher scores on this subscale indicate greater tendencies to experience height-related concerns. The NPSS-S has some advantages over the MBAS, in that it assesses cognitive, affective, and behavioral aspects of height dissatisfaction. However, this scale is also limited as it focuses exclusively on the experiences of people dissatisfied with shorter stature, and does not encompass experiences of taller individuals who wish to be shorter.

## Psychological, relational, and functional correlates of height and height dissatisfaction body image related factors

Previous research has shown that height and height dissatisfaction are related to other aspects of body image, including muscularity dissatisfaction, drive for thinness, and self-esteem. Early work by Gupta, Schork [32] found that shorter men were more likely to be preoccupied with dieting and dissatisfied with their weight, highlighting a link between height and body dissatisfaction. More recent research suggests that, similar to height, perceived pressure to conform to masculine ideals increases the risk of muscularity dissatisfaction and disordered eating among men [33].

Further studies have confirmed that height dissatisfaction is associated with muscularity dissatisfaction, general body dissatisfaction [34], and dissatisfaction with body fat [9]. Talbot and Mahlberg [9] also found that muscularity dissatisfaction and stronger idealization of muscularity predicted greater height dissatisfaction. In other words, men who were unhappy with their muscularity or who aspired to be more muscular were also more likely to be dissatisfied with their height.

Additional research by Wang [35] reported that individuals dissatisfied with being short experienced greater body image concerns, increased dissatisfaction with body fat and muscularity, and more pronounced eating disorder symptoms. One proposed explanation is that men who feel unhappy with their height, which cannot be easily changed, may attempt to compensate by focusing on alterable aspects of their appearance, such as increasing muscularity [9,32].

In relation to eating disorders, existing evidence suggests that while height dissatisfaction is modestly correlated with certain subscales of the Eating Disorder Examination Questionnaire [36], eating disorder symptoms do not appear to uniquely predict height dissatisfaction [9]. Nonetheless, observed correlations between height dissatisfaction and disordered eating behaviors highlight the need for further investigation, particularly within clinical populations.

Similarly, for women, preliminary evidence suggests that higher levels of height dissatisfaction are associated with a stronger drive for both thinness and muscularity. Notably, among taller-than-average women, drive for muscularity emerged as a prominent predictor of height dissatisfaction [6]. These findings underscore that although much of the existing research in this area has focused on individuals of shorter stature, taller women may also be vulnerable to experiencing height dissatisfaction. Like men, Favaro, Tenconi [37] found a link between low height and disordered eating symptoms amongst smaller statured women. However, this study utilized a clinical population of women with eating disorders, so the problem of causality makes it unclear whether shorter women are more likely to develop an eating disorder, or whether eating disorders, which onset at a crucial time of physical development, stunt height through malnutrition as a consequence of restricted eating practices. Talbot and Mahlberg [6] also found associations between height dissatisfaction and eating disorder symptoms in women; however, these symptoms did not uniquely predict height dissatisfaction when drive for thinness and muscularity were included in the model. This suggests that specific aspects of body image, particularly muscularity, may strengthen the relationship between height dissatisfaction and disordered eating. Adding to this, Amin, Ly [38] identified associations between dissatisfaction with shorter stature and broader concerns related to body image and weight among women.

## Self-esteem

Self-esteem refers to an individual’s subjective evaluation of their own worth [39] and is often described as the sense that one is “good enough” [40]. While several studies have explored the impact of height on self-esteem, no published research to date has specifically examined the relationship between height dissatisfaction and self-esteem.

Early work by Hensley [41] found that self-esteem was not significantly influenced by height in either men or women. In contrast, Booth [42] reported that individuals whose height deviated from the average for their gender tended to have lower self-esteem. In that study, self-consciousness played a mediating role in the relationship between height and self-esteem. More recently, Wang, Ding [43] found that shorter height was associated with lower self-esteem among adolescent girls in a Chinese sample, whereas height had little impact on self-esteem among boys. The authors suggested that cultural beauty standards may explain the gender differences, and that overall body dissatisfaction may be a more important factor influencing self-esteem than height alone. In addition, Leung, Ng [44] used immersive virtual reality to experimentally alter participants’ perceived height. They found that feeling taller led to increases in self-esteem, suggesting that height perception can influence self-evaluation in real time. Together, these findings suggest a potential link between shorter stature and lower self-esteem, although further research is needed - particularly on how dissatisfaction with height, rather than height itself, may affect self-esteem.

## Quality of life

Quality of life is a multifaceted concept, encompassing physical, emotional, social and psychological well-being [45]. Griffiths, Murray [18] outlined that height dissatisfaction had a unique association with psychological well-being and quality of life impairment amongst sexual minority men, where higher levels of height dissatisfaction were linked with lower quality of life. Another study consisting of a British adult sample identified that shorter stature was associated with a marked decline in health-related quality of life [46]. In women, Perkins, Hayes [7] found that height dissatisfaction was linked to poorer quality of life, however no associations were observed between height itself and quality of life. Similarly, Coste, Pouchot [47] found no relationship between height and health-related quality of life amongst French adults.

Amongst a sample of adolescent boys and girls, Griffiths, Murray [48] found that greater body dissatisfaction, which included a measure of height dissatisfaction, was associated with greater quality of life impairment. However, since height dissatisfaction was not utilized as an individual predictor of quality of life, it is not possible to garner height’s unique influence in this study. There is also conflicting evidence regarding this relationship in adolescents, Sommer, Daubmann [49] identified that short statured adolescents did not differ considerably from their taller counterparts in health-related quality of life.

## Psychological distress: anxiety, depression and loneliness

The association between height, anxiety, and depressive symptoms has also been investigated. Some literature investigating adult height and mortality insinuated that men who are taller are deemed at lower risk of suicide [50], as short stature is linked with psychological and cognitive factors that may elevate the likelihood of suicide [51]. However, Song, Smith [50] noted that unmeasured psychosocial and socioeconomic factors may confound this relationship, alluding to evidence that low adult height is associated with lower socioeconomic position [52] and higher behavioral risk factors [53].

Bjerkeset, Romundstad [54] conducted a study assessing the relationship between height, anxiety, depression, and suicide amongst a cohort of Norwegian men and women. Findings of this study pointed to an inverse association between height and depression and anxiety; taller individuals tended to report lower levels of depression and anxiety. Likewise, it seemed that lower suicide risk corresponded with greater height, with no major differences between males and females. Once again, the authors noted that the relationship between shorter height and higher anxiety, depression, and suicide risk was confounded by sociodemographic factors such as age and education level, as well as psychological characteristics such nervousness, calmness and mood. This aligned with the aforementioned findings of Song, Smith [50].

Related research on youth with short stature suggests that psychological difficulties are more frequently observed in clinical populations, particularly among boys who have been investigated for short stature in pediatric or endocrinological settings [55,56]. In contrast, findings are less consistent in community samples of shorter youth, where height alone does not always predict poorer psychological outcomes [49]. A similar distinction may apply in adults: it remains unclear whether short stature per se contributes directly to psychological burden, or whether distress is better explained by underlying medical or developmental conditions associated with short stature, such as growth hormone deficiency or chronic illness. Clarifying this distinction is important for understanding whether psychological outcomes reflect biological vulnerability, social stigma, or the cumulative effects of health-related challenges.

A single study has examined the relationship between height dissatisfaction and loneliness. Within a Chinese adolescent sample, Mo and Bai [57] found that greater levels of height dissatisfaction were associated with greater reported levels of loneliness, and that height dissatisfaction was a crucial predictor of loneliness. Further enquiry into this association revealed that this relationship was mediated by social anxiety. Underpinning this were feelings of fear from height-dissatisfied adolescents that they were judged through a negative lens by peers, culminating in reduced confidence and more anxiety in social situations [57].

Mo and Bai [57] found that loneliness among height-dissatisfied adolescents was not due to social exclusion from peers as a result of their stature. Instead, they posited that it appeared to stem from avoidance behavior, whereby adolescents deliberately withdrew from social situations due to their own dissatisfaction with their height.

## Romantic relationships

Height and height dissatisfaction can significantly shape social experiences, particularly in the context of romantic relationships. Traditional gender norms often influence partner preferences, with women tending to prefer men who display conventionally masculine traits, including greater height [58]. This preference is thought to stem from evolutionary beliefs that masculine features signal desirable heritable traits [59,60].

Taller men are frequently associated with qualities such as resource acquisition [61] and the ability to intimidate rivals [3], both of which may enhance their perceived attractiveness [22]. Yancey and Emerson [24] found that women often cited a desire to feel protected, secure, and feminine - as well as the wish to “look up” to their partner - as key reasons for preferring taller men. These attitudes reinforce a widely held “male-taller” norm, in which the man is expected to be taller than his female partner [3].

Empirical studies support the strength of these preferences. Salska, Frederick [3] reported that only 23% of heterosexual men and 4% of heterosexual women would accept a relationship where the woman was taller. Stulp, Buunk [62] found that women, more than men, were likely to view relationships as unacceptable when the female partner was taller. They also noted that women were more accepting of male partners who exceeded their preferred height than those who fell below it.

Similarly, Perkins, Hayes [7] found that 84% of Australian women preferred a taller romantic partner, and 57% reported they would not date someone shorter than themselves. Swami, Furnham [5] further demonstrated that both men and women tend to prefer partners who conform to this height norm, with taller men and shorter women favoring larger height differences. In contrast, shorter men and taller women typically prefer smaller height gaps and are less inclined to pursue relationships that fall outside of these expectations. Despite the persistence of these norms, some flexibility has been observed. Shorter-than-average men have shown a willingness to date women up to two inches taller [3]. Additionally, Perkins, Hayes [7] found that 56% of women in the tallest quartile of their sample were open to dating a shorter partner. These findings suggest that while cultural norms around height in romantic relationships are strong, they are not absolute.

Additionally, these findings largely reflect Western, industrialized societies where height is linked with socioeconomic and gendered ideals. Cross-cultural research suggests that height preferences and dissatisfaction are not universal; for instance, among the Himba of Namibia and the Datoga of Tanzania, preferences for tall stature are less pronounced or reversed [63,64]. Incorporating non-Western perspectives can therefore help delineate culturally specific from cross-culturally robust features of height ideals.

Further, beyond stated preferences, real-world pairings often reflect assortative mating, wherein individuals tend to choose partners with similar height [65]. This pattern suggests that social norms and biological preferences interact with opportunity structures and mutual attraction dynamics, yielding moderate but consistent positive associations between partners’ heights across cultures.

## Variability among sexual minority groups

Although research in this area is limited, emerging evidence suggests that sexual minority men may exhibit distinct patterns in height preference compared to heterosexual individuals. Valentova, Stulp [66] found that taller gay men tended to prefer taller partners, indicating that one’s own height plays a role in shaping preferences. Similarly, Griffiths, Murray [18] observed a positive correlation between a man’s actual height and the height he desired in a romantic partner. These findings suggest that the heterosexual norm of a “taller man, shorter woman” dynamic does not necessarily apply within same-sex male relationships. However, the small number of studies exploring height preferences among sexual minority populations highlights a significant gap in the literature. Much of the existing research focuses on heterosexual dynamics, leaving the nuances of attraction and body image within diverse sexual orientations underexplored. Further investigation is needed to understand the broader social, psychological, and relational implications of height preferences among sexual minority men.

## Discrimination and heightism

Beyond romantic relationships, height and height dissatisfaction also have broader social implications, including discrimination and unequal treatment - commonly referred to as heightism [67]. Griffiths, Murray [18] examined this phenomenon among sexual minority men and found shorter height was associated with higher levels of dissatisfaction and increased experiences of negative treatment. Men between 165–175 cm reported mild mistreatment based on height, while men under 165 cm experienced significantly worse treatment, illustrating the social advantage of taller stature. Similarly, amongst women, Perkins, Hayes [7] observed that shorter Australian women perceived they were treated more poorly due to their height compared to their taller counterparts.

## Workplace outcomes

Height also plays a role within professional settings. Taller individuals are often

perceived as more persuasive [68] and more likely to be viewed as leaders [69]. These perceptions are particularly influential in workplace environments where leadership and confidence are valued [61].

While job performance may not be linked to height, perceived competence is. Judge and Cable [61] found strong positive correlations between height and income, with taller individuals earning more on average over their careers. For instance, someone 182 cm tall was predicted to earn approximately $166, 000 USD (approximately $258,938 USD today) more across a 30-year career than someone who was 165 cm tall. These findings held when controlling for gender, suggesting that both taller men and women benefit financially.

Additional studies support this trend. Taller women are often viewed as having greater authority in the workplace [70], and more likely to have partners with higher occupational statuses [71]. These findings reinforce the broader patterns that height influences perceived status and success, irrespective of objective performance.

## Theoretical perspectives of height dissatisfaction

In this section we have applied height dissatisfaction to established theoretical frameworks, including evolutionary psychology and sociocultural perspectives such as the Tripartite Influence Model. While these frameworks offer useful lenses for understanding potential origins and maintenance of height dissatisfaction, their application to height remains largely untested empirically. Nonetheless, they provide valuable conceptual foundations to guide future research aimed at uncovering the mechanics behind height dissatisfaction and informing intervention strategies.

### Evolutionary perspectives

Evolutionary psychology provides a compelling framework for understanding the enduring social and reproductive significance of height, particularly in relation to dominance, leadership, and mate selection. Across ancestral environments, stature likely served as a visible cue to formidability and resource-holding potential—qualities that facilitated survival, group coordination, and access to mates. Height may have conferred advantages in physical contests and coalition formation, leading to evolved cognitive mechanisms that link tallness with authority and competence [59,72].

#### Dominance and status.

Height is closely intertwined with perceptions of dominance, status, and success. Taller individuals are often perceived as more assertive, persuasive, and capable of leadership [21,61,73]. These social perceptions may reflect ancestral adaptations in which physical stature signalled both the capacity to defend and to provide. Modern evidence supports these associations: height correlates positively with educational attainment [14,74,75], occupational status [76], and income [77–81]. These findings suggest that societal structures continue to reinforce the adaptive advantages once afforded by taller stature, even in contexts far removed from ancestral selection pressures.

#### Leadership and coalition-building.

Evolutionary leadership theory posits that early human groups favoured leaders who appeared physically capable and authoritative, as visible cues of strength and coordination facilitated group cohesion in collective action scenarios [59,72,73]. Empirical work supports a “height–leadership advantage,” with taller individuals more frequently perceived as natural leaders across professional and social domains [59,73,82]. This preference likely persists because it taps into ancient heuristics that associated physical stature with protective capacity and effective guidance in intergroup conflict or resource acquisition.

#### Mate preferences and reproductive fitness.

From a reproductive standpoint, female preferences for taller males may have evolved because height signalled health, protection, and superior genetic fitness [61,73,83]. Height is a highly heritable trait, and ancestral women may have preferred taller partners to enhance offspring survival prospects and social standing. Empirical evidence supports this: taller men are perceived as more attractive, dominant, and socially competent across many cultures [3,5]. Conversely, female height preferences tend to reflect both evolutionary and cultural forces—while shorter stature aligns with traditional femininity ideals, taller women may experience social tension when exceeding normative gender expectations [6,23].

#### Implications for height dissatisfaction.

When viewed through this lens, height dissatisfaction may emerge from a mismatch between evolved psychological preferences and individual reality. Men who fall below culturally and evolutionarily ideal standards of tallness may experience diminished self-esteem or status anxiety, whereas taller women may internalize negative feedback for deviating from feminine norms. These processes underscore how ancestral selection pressures on height and dominance continue to shape modern psychosocial functioning and self-evaluation.

### Sociocultural and media influence perspectives

While evolutionary theory offers insight into the ancestral origins of height-related preferences, sociocultural perspectives may provide a more comprehensive understanding of how height dissatisfaction develops and is maintained in modern society, The predominant framework in body image research is the Tripartite Influence model [84], which suggests that body dissatisfaction- potentially including dissatisfaction with height- is shaped by three primary sociocultural influences: peers, parents, and media. These agents exert influence directly, through comments and feedback, and indirectly, through modelling and reinforcement of appearance ideals. Central to this process is Social Comparison Theory [85], which proposes that individuals evaluate themselves by comparing their physical characteristics to those of others. Through these pathways, individuals may come to internalize societal standards, including those related to height, and engage in appearance-based comparisons, contributing to dissatisfaction when personal attributes fall short of perceived ideals [86].

Although height is less frequently studied than body fat or muscularity, it remains a highly visible and socially meaningful attribute. Parental influences may contribute to height dissatisfaction, particularly through appearance-related commentary or teasing. For example, negative parental input has been linked to increased appearance comparison and dissatisfaction [87,88]. Among boys, parental encouragement to lose weight has been associated with a range of body image concerns, including body dissatisfaction, drive for muscularity, and binge eating [89]; while height is not always explicitly addressed, such feedback may reinforce the idea that one’s body (including height) does not meet valued standards. Notably, parental teasing has not shown a consistent relationship with body dissatisfaction amongst boys, suggesting that other social influences may be more salient in height-related concerns [89].

Peer influence is particularly relevant to height dissatisfaction, especially during adolescence, when comparisons to same-gender peers can shape body image concerns. Teasing about height or exclusion due to perceived physical inadequacy can directly contribute to negative self-evaluation. In parallel, media portrayal consistently reinforces narrow ideals of the male body, in which tallness is associated with masculinity, strength, and attractiveness [4]. Among Chinese college students, greater consumption of media depicting idealized body types was linked to appearance comparisons and body dissatisfaction [90]. Similarly, research on social media use shows that time spent on platforms like Facebook is associated with increased appearance comparison and decreased appearance satisfaction [91,92], patterns that may extend to height dissatisfaction when users are exposed to tall, idealized bodies.

Although the Tripartite Influence Model has not been directly tested in relation to height dissatisfaction, its core mechanisms are applicable. Height is a salient aspect of appearance that is linked to broader constructs such as muscularity, dominance and social desirability- particularly in men [34]. It is therefore plausible that the same sociocultural influences contributing to dissatisfaction with weight and body shape may also shape dissatisfaction with height. Among women, the model has primarily explained dissatisfaction

with fatness and thinness, but in cultural contexts where shorter female stature is idealized, these pressures may similarly result in height-related dissatisfaction among taller women who feel they deviate from these appearance norms.

## Clinical implications

Height dissatisfaction is an important but often overlooked component of body image concerns, particularly for individuals of shorter stature, who are more vulnerable to experiencing height dissatisfaction due to the strong relationship between shorter height and negative self-perceptions. Clinicians should be mindful that height dissatisfaction frequently co-occurs with other body image-related factors such as muscularity dissatisfaction [33], drive for thinness [6], and body fat dissatisfaction [9]. This constellation of concerns may contribute to compensatory behaviors (like excessive focus on muscularity) that may increase the risk of disordered eating and related psychopathology. Given its impact on self-esteem and quality of life, height dissatisfaction warrants inclusion in comprehensive assessments of body image and psychological well-being. Therapeutic approaches that target negative self-evaluations related to height, promote adaptive coping, and address internalized sociocultural standards of appearance may be particularly beneficial.

Ultimately, integrating height dissatisfaction into clinical formulations supports a more holistic understanding of clients’ body image struggles and related psychosocial impairments. While further research is needed to develop empirically validated interventions specifically targeting height dissatisfaction, current evidence underscores the importance of addressing this issue to improve self-esteem, reduce psychological distress and enhance social functioning among those affected.

## Conclusions and future recommendations

Although research on height and height dissatisfaction has expanded in recent years, this area of body image research is still in its infancy, with several gaps that warrant further investigation. First, as our paper is a narrative review, it is subject to several methodological constraints inherent to this format [93]. Consequently, the inclusion of literature may be influenced by publication bias, and findings should be interpreted as indicative rather than conclusive. In line with recommendations on constraints on generality [94], we encourage readers to consider that many cited studies draw from Western samples and may not generalize across cultures or contexts. Second, most studies on height and height dissatisfaction have been cross-sectional, limiting understanding of how height dissatisfaction changes over time. Longitudinal research is essential to clarify causal pathways and the temporal relationship between height dissatisfaction and psychological outcomes such as anxiety, depression, and body image disturbance. Third, there is a pressing need for the development and validation of a comprehensive scale specifically designed to measure height dissatisfaction. As discussed, existing measures fail to capture the complexity of height-related concerns, as well as associated cognitive, affective and behavioral factors. A multidimensional instrument would enable more precise identification of individuals experiencing clinically significant height dissatisfaction and enhance research consistency. Fourth, much of the existing literature focuses on shorter men, yet height dissatisfaction may also affect taller individuals, particularly women, who may experience pressures related to deviations from culturally ideal female height. Future research should explore the nuanced experiences of height dissatisfaction across genders, sexual orientations, and cultures to provide a more inclusive understanding. Fifth, a key limitation of our review is its sole focus on adult populations. Although height dissatisfaction and its psychosocial correlates are well-documented in adults, these concerns may originate during childhood or, more commonly, in the pubertal years in step with other forms of body dissatisfaction [95]. Indeed, it is in the pubertal years when height differences become more salient [96] and social comparison processes intensify [97]. Future research should therefore examine the developmental trajectory of height dissatisfaction, including how early experiences of teasing, peer comparison, and identity formation contribute to later body image outcomes. Additionally, future research should distinguish between the psychological consequences of short stature itself and those arising from medical or developmental diagnoses associated with reduced growth. Understanding whether distress reflects social stigma, internalized appearance concerns, or chronic health burden will help clarify the mechanisms linking stature to mental health outcomes in both youth and adult populations. Sixth, there is limited empirical evaluation of theoretical models applied to height dissatisfaction, particularly sociocultural frameworks such as the Tripartite Influence Model. Testing these models can clarify mechanisms by which height dissatisfaction arises and is maintained, informing targeted intervention strategies.

Seventh, many foundational studies in this field were conducted over a decade ago, during a period when societal standards, norms, and media exposure differed considerably from today. Given the evolving nature of appearance ideals that are shaped by digital media, shifting gender roles, and increased representation of diverse bodies, it is critical that future research examines whether previously identified trends in height preference and dissatisfaction remain consistent in contemporary contexts. Longitudinal and cohort-based research could help determine whether societal changes have influenced how height is perceived and experienced across time. Last, intervention research is needed to develop and evaluate psychological treatments that address height dissatisfaction and its related psychological impacts. Interventions could focus on reducing maladaptive social comparisons, challenging internalized height ideals, and managing avoidance behaviors. Addressing these priorities will deepen our understanding of height dissatisfaction and support the development of effective prevention and treatment approaches tailored to this unique aspect of body image.

## References

[1] Medeiros A. Heightened expectations: The rise of the human growth hormone industry in America. University of Alabama Press. 2016. vol. 3.

[2] Batres C, Re DE, Perrett DI. Influence of Perceived Height, Masculinity, and Age on Each Other and on Perceptions of Dominance in Male Faces. Perception. 2015;44(11):1293–309. doi: 10.1177/0301006615596898 26562897

[3] Salska I, Frederick DA, Pawlowski B, Reilly AH, Laird KT, Rudd NA. Conditional mate preferences: Factors influencing preferences for height. Personality and Individual Differences. 2008;44(1):203–15. doi: 10.1016/j.paid.2007.08.008

[4] Ridgeway RT, Tylka TL. College Men’s Perceptions of Ideal Body Composition and Shape. Psychology of Men & Masculinity. 2005;6(3):209–20. doi: 10.1037/1524-9220.6.3.209

[5] Swami V, Furnham A, Balakumar N, Williams C, Canaway K, Stanistreet D. Factors influencing preferences for height: A replication and extension. Personality and Individual Differences. 2008;45(5):395–400. doi: 10.1016/j.paid.2008.05.012

[6] Talbot D, Mahlberg J. An examination of the relationship between height, height dissatisfaction, drive for thinness and muscularity, and eating disorder symptoms in north American women. Curr Psychol. 2024;43(29):24346–54. doi: 10.1007/s12144-024-06108-z

[7] Perkins T, Hayes S, Talbot D. Shorter Women Are More Dissatisfied with Their Height: An Exploration of Height Dissatisfaction in Australian Women. Obesities. 2021;1(3):189–99. doi: 10.3390/obesities1030017

[8] Bergeron D, Tylka TL. Support for the uniqueness of body dissatisfaction from drive for muscularity among men. Body Image. 2007;4(3):288–95. doi: 10.1016/j.bodyim.2007.05.002 18089275

[9] Talbot D, Mahlberg J. Exploration of height dissatisfaction, muscle dissatisfaction, body ideals, and eating disorder symptoms in men. J Am Coll Health. 2023;71(1):18–23. doi: 10.1080/07448481.2021.1877143 33577425

[10] Macgregor S, Cornes BK, Martin NG, Visscher PM. Bias, precision and heritability of self-reported and clinically measured height in Australian twins. Hum Genet. 2006;120(4):571–80. doi: 10.1007/s00439-006-0240-z 16933140

[11] Perola M, Sammalisto S, Hiekkalinna T, Martin NG, Visscher PM, Montgomery GW, et al. Combined genome scans for body stature in 6,602 European twins: evidence for common Caucasian loci. PLoS Genet. 2007;3(6):e97. doi: 10.1371/journal.pgen.0030097 17559308 PMC1892350

[12] McEvoy BP, Visscher PM. Genetics of human height. Econ Hum Biol. 2009;7(3):294–306. doi: 10.1016/j.ehb.2009.09.005 19818695

[13] Chen X, Kuja-Halkola R, Rahman I, Arpegård J, Viktorin A, Karlsson R, et al. Dominant Genetic Variation and Missing Heritability for Human Complex Traits: Insights from Twin versus Genome-wide Common SNP Models. Am J Hum Genet. 2015;97(5):708–14. doi: 10.1016/j.ajhg.2015.10.004 26544805 PMC4667127

[14] Silventoinen K. Determinants of variation in adult body height. J Biosoc Sci. 2003;35(2):263–85. doi: 10.1017/s0021932003002633 12664962

[15] Komlos J, Baur M. From the tallest to (one of) the fattest: the enigmatic fate of the American population in the 20th century. Econ Hum Biol. 2004;2(1):57–74. doi: 10.1016/j.ehb.2003.12.006 15463993

[16] Hatton TJ. How have Europeans grown so tall?. Oxford Economic Papers. 2014;66(2):349–72.

[17] Chen H, Jackson T, Huang X. The Negative Physical Self Scale: Initial development and validation in samples of Chinese adolescents and young adults. Body Image. 2006;3(4):401–12. doi: 10.1016/j.bodyim.2006.07.005 18089244

[18] Griffiths S, Murray SB, Medeiros A, Blashill AJ. The tall and the short of it: An investigation of height ideals, height preferences, height dissatisfaction, heightism, and height-related quality of life impairment among sexual minority men. Body Image. 2017;23:146–54. doi: 10.1016/j.bodyim.2017.10.001 29031097

[19] Berger JL, Addis ME, Green JD, Mackowiak C, Goldberg V. Men’s reactions to mental health labels, forms of help-seeking, and sources of help-seeking advice. Psychology of Men & Masculinity. 2013;14(4):433–43. doi: 10.1037/a0030175

[20] Rahman O, Navarro HD. Perceptions of men’s height and self-objectification: Critical social perspectives across gender and age groups. Trends in Psychology. 2023;:1–18.

[21] Melamed T. Personality correlates of physical height. Personality and Individual Differences. 1992;13(12):1349–50. doi: 10.1016/0191-8869(92)90179-s

[22] Pawlowski B, Jasienska G. Women’s preferences for sexual dimorphism in height depend on menstrual cycle phase and expected duration of relationship. Biol Psychol. 2005;70(1):38–43. doi: 10.1016/j.biopsycho.2005.02.002 16038772

[23] Thesander M. The feminine ideal. Reaktion Books. 1997.

[24] Yancey G, Emerson MO. Does Height Matter? An Examination of Height Preferences in Romantic Coupling. Journal of Family Issues. 2014;37(1):53–73. doi: 10.1177/0192513x13519256

[25] Rayner J-A, Pyett P, Astbury J. The medicalisation of “tall” girls: A discourse analysis of medical literature on the use of synthetic oestrogen to reduce female height. Soc Sci Med. 2010;71(6):1076–83. doi: 10.1016/j.socscimed.2010.06.026 20678835

[26] Barnard ND, Scialli AR, Bobela S. The current use of estrogens for growth-suppressant therapy in adolescent girls. J Pediatr Adolesc Gynecol. 2002;15(1):23–6. doi: 10.1016/s1083-3188(01)00135-8 11888806

[27] Jacobi L, Cash TF. In Pursuit of the Perfect Appearance: Discrepancies Among Self‐Ideal Percepts of Multiple Physical Attributes 1. J Applied Social Pyschol. 1994;24(5):379–96. doi: 10.1111/j.1559-1816.1994.tb00588.x

[28] Tylka TL, Bergeron D, Schwartz JP. Development and psychometric evaluation of the Male Body Attitudes Scale (MBAS). Body Image. 2005;2(2):161–75. doi: 10.1016/j.bodyim.2005.03.001 18089184

[29] Ryan TA, Morrison TG, Roddy S, McCutcheon J. Psychometric properties of the Revised Male Body Attitudes Scale among Irish men. Body Image. 2011;8(1):64–9. doi: 10.1016/j.bodyim.2010.10.004 21095167

[30] Blashill AJ, Vander Wal JS. The Male Body Attitudes Scale: A confirmatory factor analysis with a sample of gay men. Body Image. 2009;6(4):322–5. doi: 10.1016/j.bodyim.2009.07.004 19674947

[31] Costello AB, Osborne J. Best practices in exploratory factor analysis: Four recommendations for getting the most from your analysis. Practical Assessment, Research, and Evaluation. 2005;10(1).

[32] Gupta MA, Schork NJ, Dhaliwal JS. Stature, drive for thinness and body dissatisfaction: a study of males and females from a non clinical sample. Can J Psychiatry. 1993;38(1):59–61. doi: 10.1177/070674379303800115 8448724

[33] Griffiths S, Murray SB, Touyz S. Extending the masculinity hypothesis: An investigation of gender role conformity, body dissatisfaction, and disordered eating in young heterosexual men. Psychology of Men & Masculinity. 2015;16(1):108–14. doi: 10.1037/a0035958

[34] O’Gorman B, Sheffield J, Griffiths S. Does masculinity moderate the relationship of height with height dissatisfaction? Findings from an Internet forum for short statured men. Body Image. 2019;31:112–9. doi: 10.1016/j.bodyim.2019.09.002 31569064

[35] Wang Y-H, Wang Y-L, Ly M, Nichol M, Misener K, Libben M. Factorial validity, reliability, and measurement invariance of the Negative Physical Self Scale in a sample of men residing in North America. Psychol Assess. 2022;34(11):1036–46. doi: 10.1037/pas0001165 36074611

[36] Fairburn CG. Cognitive behavior therapy and eating disorders. Guilford Press. 2008.

[37] Favaro A, Tenconi E, Degortes D, Soave M, Zanetti T, Nardi MT, et al. Association between low height and eating disorders: cause or effect?. Int J Eat Disord. 2007;40(6):549–53. doi: 10.1002/eat.20409 17584869

[38] Amin S, Ly M, Misener K, Brown N, Libben M. Validation of the translated Negative Physical Self Scale in a sample of Asian women living in Canada. PLoS One. 2024;19(5):e0301184. doi: 10.1371/journal.pone.0301184 38696442 PMC11065207

[39] Donnellan MB, Kenny DA, Trzesniewski KH, Lucas RE, Conger RD. Using Trait-State Models to Evaluate the Longitudinal Consistency of Global Self-Esteem From Adolescence to Adulthood. J Res Pers. 2012;46(6):634–45. doi: 10.1016/j.jrp.2012.07.005 23180899 PMC3501685

[40] Orth U, Robins RW. The Development of Self-Esteem. Curr Dir Psychol Sci. 2014;23(5):381–7. doi: 10.1177/0963721414547414

[41] Hensley WE. Gender, Self-Esteem and Height. Percept Mot Skills. 1983;56(1):235–8. doi: 10.2466/pms.1983.56.1.235

[42] Booth ND. The Relationship between Height and Self-Esteem and the Mediating Effect of Self-Consciousness. The Journal of Social Psychology. 1990;130(5):609–17. doi: 10.1080/00224545.1990.9922952

[43] Wang D, Ding S, Wang J, Gong Y, Xu X. Gender differences in height, weight and BMI on self-esteem among rural school-aged children in China. Theory Clin Pract Pediatr. 2017;1(1). doi: 10.25082/tcpp.2017.01.003

[44] .44. Leung GY, Ng AK, Lau HY. Effect of height perception on state self-esteem and cognitive performance in virtual reality. In: International Conference on Human-Computer Interaction. Springer. 2021.

[45] Rodrigues C, Silva M, Cerejo R, Rodrigues R, Sousa L, Trigo C, et al. Quality of life among adults with repaired tetralogy of fallot: A literature review. Rev Port Cardiol (Engl Ed). 2021;40(12):969–74. doi: 10.1016/j.repce.2021.11.016 34922706

[46] Christensen TL, Djurhuus CB, Clayton P, Christiansen JS. An evaluation of the relationship between adult height and health-related quality of life in the general UK population. Clin Endocrinol (Oxf). 2007;67(3):407–12. doi: 10.1111/j.1365-2265.2007.02901.x 17573903

[47] Coste J, Pouchot J, Carel J-C. Height and health-related quality of life: a nationwide population study. J Clin Endocrinol Metab. 2012;97(9):3231–9. doi: 10.1210/jc.2012-1543 22745240

[48] Griffiths S, Murray SB, Bentley C, Gratwick-Sarll K, Harrison C, Mond JM. Sex Differences in Quality of Life Impairment Associated With Body Dissatisfaction in Adolescents. J Adolesc Health. 2017;61(1):77–82. doi: 10.1016/j.jadohealth.2017.01.016 28389062

[49] Sommer R, Daubmann A, Quitmann J, Ravens-Sieberer U, Bullinger M. Understanding the impact of statural height on health-related quality of life in German adolescents: a population-based analysis. Eur J Pediatr. 2015;174(7):875–82. doi: 10.1007/s00431-014-2480-6 25535173

[50] Song Y-M, Smith GD, Sung J. Adult height and cause-specific mortality: a large prospective study of South Korean men. Am J Epidemiol. 2003;158(5):479–85. doi: 10.1093/aje/kwg173 12936903

[51] Jiang GX, Rasmussen F, Wasserman D. Short stature and poor psychological performance: risk factors for attempted suicide among Swedish male conscripts. Acta Psychiatr Scand. 1999;100(6):433–40. doi: 10.1111/j.1600-0447.1999.tb10893.x 10626921

[52] Leon DA, Smith GD, Shipley M, Strachan D. Adult height and mortality in London: early life, socioeconomic confounding, or shrinkage?. J Epidemiol Community Health. 1995;49(1):5–9. doi: 10.1136/jech.49.1.5 7707006 PMC1060066

[53] Smith GD, Phillips AN, Neaton JD. Smoking as “independent” risk factor for suicide: illustration of an artifact from observational epidemiology?. Lancet. 1992;340(8821):709–12. doi: 10.1016/0140-6736(92)92242-8 1355809

[54] Bjerkeset O, Romundstad P, Evans J, Gunnell D. Association of adult body mass index and height with anxiety, depression, and suicide in the general population: the HUNT study. Am J Epidemiol. 2008;167(2):193–202. doi: 10.1093/aje/kwm280 17981889

[55] Attanasio AF, Shavrikova EP, Blum WF, Shalet SM. Quality of life in childhood onset growth hormone-deficient patients in the transition phase from childhood to adulthood. J Clin Endocrinol Metab. 2005;90(8):4525–9. doi: 10.1210/jc.2005-0439 15899956

[56] Abe S, Okumura A, Mukae T, Nakazawa T, Niijima S-I, Yamashiro Y, et al. Depressive tendency in children with growth hormone deficiency. J Paediatr Child Health. 2009;45(11):636–40. doi: 10.1111/j.1440-1754.2009.01586.x 19845844

[57] Mo Q-ZL, Bai B-Y. Height dissatisfaction and loneliness among adolescents: the chain mediating role of social anxiety and social support. Curr Psychol. 2022;:1–9. doi: 10.1007/s12144-022-03855-9 36277262 PMC9579572

[58] Johnston VS, Hagel R, Franklin M, Fink B, Grammer K. Male facial attractiveness: evidence for hormone-mediated adaptive design. Evolution and Human Behavior. 2001;22(4):251–67. doi: 10.1016/s1090-5138(01)00066-6

[59] Blaker NM, Rompa I, Dessing IH, Vriend AF, Herschberg C, van Vugt M. The height leadership advantage in men and women: Testing evolutionary psychology predictions about the perceptions of tall leaders. Group Processes & Intergroup Relations. 2013;16(1):17–27. doi: 10.1177/1368430212437211

[60] Sell A, Cosmides L, Tooby J, Sznycer D, von Rueden C, Gurven M. Human adaptations for the visual assessment of strength and fighting ability from the body and face. Proc Biol Sci. 2009;276(1656):575–84. doi: 10.1098/rspb.2008.1177 18945661 PMC2664345

[61] Judge TA, Cable DM. The effect of physical height on workplace success and income: preliminary test of a theoretical model. J Appl Psychol. 2004;89(3):428–41. doi: 10.1037/0021-9010.89.3.428 15161403

[62] Stulp G, Buunk AP, Pollet TV. Women want taller men more than men want shorter women. Personality and Individual Differences. 2013;54(8):877–83. doi: 10.1016/j.paid.2012.12.019

[63] Sorokowski P, Butovskaya ML. Height preferences in humans may not be universal: evidence from the Datoga people of Tanzania. Body Image. 2012;9(4):510–6. doi: 10.1016/j.bodyim.2012.07.002 22871368

[64] Sorokowski P, Sorokowska A, Fink B, Mberira M. Variable Preferences for Sexual Dimorphism in Stature (SDS) Might Not Be Universal. Journal of Cross-Cultural Psychology. 2011;43(1):32–7. doi: 10.1177/0022022110395140

[65] Stulp G, Simons MJP, Grasman S, Pollet TV. Assortative mating for human height: A meta-analysis. Am J Hum Biol. 2017;29(1):e22917. doi: 10.1002/ajhb.22917 27637175 PMC5297874

[66] Valentova JV, Stulp G, Třebický V, Havlíček J. Preferred and actual relative height among homosexual male partners vary with preferred dominance and sex role. PLoS One. 2014;9(1):e86534. doi: 10.1371/journal.pone.0086534 24466136 PMC3899263

[67] Feldman, SD, *The presentation of shortness in everyday life. Height and heightism in American society: toward a sociology of stature.* Life styles: Diversity in American society, 1975: p. 427–42.

[68] Young TJ, French LA. Height and perceived competence of US presidents. Percept Mot Skills. 1996;82(3 Pt 1):1002. doi: 10.1177/003151259608200301 8774043

[69] Higham PA, Carment DW. The rise and fall of politicians: The judged heights of Broadbent, Mulroney and Turner before and after the 1988 Canadian federal election. Canadian Journal of Behavioural Science / Revue canadienne des sciences du comportement. 1992;24(3):404–9. doi: 10.1037/h0078723

[70] Gawley T, Perks T, Curtis J. Height, Gender, and Authority Status at Work: Analyses for a National Sample of Canadian Workers. Sex Roles. 2008;60(3–4):208–22. doi: 10.1007/s11199-008-9520-5

[71] Murasko JE. Height, marriage, and partner characteristics for women in low- and middle-income countries. Econ Hum Biol. 2020;38:100876. doi: 10.1016/j.ehb.2020.100876 32485639

[72] Spisak BR, Nicholson N, van Vugt M. Leadership in organizations: An evolutionary perspective. Evolutionary psychology in the business sciences. Springer. 2011. p. 165–90.

[73] Murray GR, Schmitz JD. Caveman Politics: Evolutionary Leadership Preferences and Physical Stature. Social Science Quarterly. 2011;92(5):1215–35. doi: 10.1111/j.1540-6237.2011.00815.x

[74] Magnusson PKE, Rasmussen F, Gyllensten UB. Height at age 18 years is a strong predictor of attained education later in life: cohort study of over 950,000 Swedish men. Int J Epidemiol. 2006;35(3):658–63. doi: 10.1093/ije/dyl011 16446353

[75] Cinnirella F, Piopiunik M, Winter J. Why does height matter for educational attainment? Evidence from German children. Econ Hum Biol. 2011;9(4):407–18. doi: 10.1016/j.ehb.2011.04.006 21616729

[76] Heineck G. Up in the Skies? The Relationship between Body Height and Earnings in Germany. Labour. 2005;19(3):469–89. doi: 10.1111/j.1467-9914.2005.00302.x

[77] Lundborg P, Nystedt P, Rooth D-O. Height and Earnings: The Role of Cognitive and Noncognitive Skills. J Human Resources. 2014;49(1):141–66. doi: 10.3368/jhr.49.1.141

[78] Kortt M, Leigh A. Does size matter in Australia?. Economic Record. 2010;86(272):71–83.

[79] Case A, Paxson C. Stature and status: Height, ability, and labor market outcomes. J Polit Econ. 2008;116(3):499–532. doi: 10.1086/589524 19603086 PMC2709415

[80] Böckerman P, Vainiomäki J. Stature and life-time labor market outcomes: Accounting for unobserved differences. Labour Economics. 2013;24:86–96. doi: 10.1016/j.labeco.2013.06.003

[81] Persico N, Postlewaite A, Silverman D. The Effect of Adolescent Experience on Labor Market Outcomes: The Case of Height. Journal of Political Economy. 2004;112(5):1019–53. doi: 10.1086/422566

[82] Stulp G, Buunk AP, Verhulst S, Pollet TV. Tall claims? Sense and nonsense about the importance of height of US presidents. The Leadership Quarterly. 2013;24(1):159–71. doi: 10.1016/j.leaqua.2012.09.002

[83] Stulp G, Barrett L. Evolutionary perspectives on human height variation. Biol Rev Camb Philos Soc. 2016;91(1):206–34. doi: 10.1111/brv.12165 25530478

[84] Thompson JK, Heinberg LJ, Altabe MN, Tantlee-Dunn. Exacting beauty: theory, assessment, and treatment of body image disturbance. Thomson JK, Heinberg LJ, Altabe MN, Tantlee-Dunn. Exacting beauty: theory, assessment, and treatment of body image disturbance. Washington, DC: American Psychological Association. 1999.

[85] Festinger L. A Theory of Social Comparison Processes. Human Relations. 1954;7(2):117–40. doi: 10.1177/001872675400700202

[86] Keery H, van den Berg P, Thompson JK. An evaluation of the Tripartite Influence Model of body dissatisfaction and eating disturbance with adolescent girls. Body Image. 2004;1(3):237–51. doi: 10.1016/j.bodyim.2004.03.001 18089156

[87] Bailey SD, Ricciardelli LA. Social comparisons, appearance related comments, contingent self-esteem and their relationships with body dissatisfaction and eating disturbance among women. Eat Behav. 2010;11(2):107–12. doi: 10.1016/j.eatbeh.2009.12.001 20188294

[88] Levine MP, Smolak L. Primary prevention of body image disturbances and disordered eating in childhood and early adolescence. 2001.

[89] Rodgers R, Chabrol H. Parental attitudes, body image disturbance and disordered eating amongst adolescents and young adults: a review. Eur Eat Disord Rev. 2009;17(2):137–51. doi: 10.1002/erv.907 19130467

[90] Shen J, Chen J, Tang X, Bao S. The effects of media and peers on negative body image among Chinese college students: a chained indirect influence model of appearance comparison and internalization of the thin ideal. J Eat Disord. 2022;10(1):49. doi: 10.1186/s40337-022-00575-0 35413877 PMC9006462

[91] Holland G, Tiggemann M. A systematic review of the impact of the use of social networking sites on body image and disordered eating outcomes. Body Image. 2016;17:100–10. doi: 10.1016/j.bodyim.2016.02.008 26995158

[92] Saiphoo AN, Vahedi Z. A meta-analytic review of the relationship between social media use and body image disturbance. Computers in Human Behavior. 2019;101:259–75. doi: 10.1016/j.chb.2019.07.028

[93] Siddaway AP, Wood AM, Hedges LV. How to Do a Systematic Review: A Best Practice Guide for Conducting and Reporting Narrative Reviews, Meta-Analyses, and Meta-Syntheses. Annu Rev Psychol. 2019;70:747–70. doi: 10.1146/annurev-psych-010418-102803 30089228

[94] Simons DJ, Shoda Y, Lindsay DS. Constraints on Generality (COG): A Proposed Addition to All Empirical Papers. Perspect Psychol Sci. 2017;12(6):1123–8. doi: 10.1177/1745691617708630 28853993

[95] Foster L, Lundh L-G, Daukantaité D. Disordered eating in a 10-year perspective from adolescence to young adulthood: Stability, change, and body dissatisfaction as a predictor. Scand J Psychol. 2024;65(1):32–41. doi: 10.1111/sjop.12950 37491950

[96] Haas JD, Campirano F. Interpopulation variation in height among children 7 to 18 years of age. Food Nutr Bull. 2006;27(4 Suppl Growth Standard):S212-23. doi: 10.1177/15648265060274S505 17361658

[97] Scully M, Swords L, Nixon E. Social comparisons on social media: online appearance-related activity and body dissatisfaction in adolescent girls. Ir J Psychol Med. 2023;40(1):31–42. doi: 10.1017/ipm.2020.93 32912367

